# Multi-Species Synbiotic Supplementation After Antibiotics Promotes Recovery of Microbial Diversity and Function, and Increases Gut Barrier Integrity: A Randomized, Placebo-Controlled Trial

**DOI:** 10.3390/antibiotics15020138

**Published:** 2026-01-30

**Authors:** Brooke A. Napier, Jessica R. Allegretti, Paul Feuerstadt, Colleen R. Kelly, Nicholas W. Van Hise, Ralf Jäger, Gerrit A. Stuivenberg, Zain Kassam, Gregor Reid

**Affiliations:** 1 Seed Health, Inc., Venice, CA 90291, USA; 2 Division of Gastroenterology, Hepatology, and Endoscopy, Brigham and Women’s Hospital, Harvard Medical School, Boston, MA 02115, USA; 3 Department of Digestive Diseases, Yale University School of Medicine, New Haven, CT 06510, USA; 4 Department of Gastroenterology, PACT Gastroenterology Center, Hamden, CT 06518, USA; 5 Metro Infectious Disease Consultants, Burr Ridge, IL 60527, USA; 6 Increnovo LLC, Whitefish Bay, WI 53217, USA; 7 Canadian R&D Centre for Human Microbiome and Probiotics, Lawson Health Research Institute, London, ON N6A 4V2, Canada; 8 Departments of Microbiology and Immunology and Surgery, Western University, London, ON N6A 3K7, Canada

**Keywords:** antibiotic-associated dysbiosis, gut microbiome diversity, Urolithin A, probiotic, synbiotic

## Abstract

**Background**: Antibiotics are essential for treating infections; however, they disrupt the microbiome and key microbiome-dependent functions. Clinical evidence is mixed for probiotic supplementation following antibiotics due to product heterogeneity and inconsistencies in evaluating biological mechanisms that drive clinical consequences. Accordingly, this study investigates the effects of a multi-species synbiotic on gut microbiome composition and function, and gut barrier integrity, during and following antibiotics. **Methods**: In a randomized, placebo-controlled trial designed to assess proof-of-mechanism, healthy adult participants received a daily synbiotic (53.6 billion AFU multi-species probiotic and 400 mg Indian pomegranate extract; DS-01) or matching placebo for 91 days. All participants also received ciprofloxacin (500 mg orally twice daily) and metronidazole (500 mg orally three times daily) for the first 7 days. Samples were collected at baseline and Days 7, 14, 49, and 91. Endpoints included fecal microbiome composition, fecal acetate and butyrate levels, urinary Urolithin A (UroA), serum *p*-cresol sulfate (pCS), gut barrier integrity, and safety. **Results**: The multi-species synbiotic significantly increased the alpha-diversity of *Bifidobacterium* and *Lactobacillus* at all timepoints compared to placebo, including short-term (Day 7, *p* < 0.0001) and end-of-study (Day 91, *p* < 0.001). The multi-species synbiotic enhanced recovery of native beneficial microbes, including butyrate-producing species and a novel *Oscillospiraceae* species (UMGS1312 sp900550625, *p* < 0.001). Beneficial microbiome-dependent metabolites increased, including fecal butyrate (119%, *p* < 0.05), fecal acetate (62%, *p* < 0.01), and UroA (13,008%, *p* < 0.05), whereas detrimental metabolite pCS decreased (68%, *p* < 0.05) compared to placebo. Functionally, the multi-species synbiotic improved gut barrier integrity rapidly (Day 7; 305%, *p* < 0.05) and over the long-term (Day 91; 161%, *p* < 0.05) compared to placebo. **Conclusions**: During and after antibiotics, this multi-species synbiotic promotes recovery of gut microbiome diversity and native beneficial microbes, microbiome metabolite recovery, and gut barrier function, all of which underpin antibiotic-associated gastrointestinal symptoms.

## 1. Introduction

Antibiotics remain indispensable for the treatment of acute bacterial infections and are among the most frequently prescribed drug classes worldwide, with more than 230 million outpatient prescriptions written annually in the United States alone [1] and continued growth projected globally [2]. Despite their therapeutic necessity, broad-spectrum antibiotics induce rapid and often profound disruption of the gut microbiome, resulting in loss of taxonomic diversity, depletion of keystone microbes, and suppression of microbiome-dependent metabolic pathways that support intestinal homeostasis.

Clinically, this acute ecological injury commonly manifests as antibiotic-associated gastrointestinal (GI) symptoms, including diarrhea, bloating, abdominal discomfort, and altered bowel habits [3,4]. These symptoms arise not only from shifts in luminal osmotic balance, but also from antibiotic-induced suppression of microbiome-regulated epithelial transporters, impaired fermentation of dietary substrates, and loss of barrier-protective metabolites [5,6]. In many individuals, recovery of these biological functions continues beyond the period of antibiotic administration, contributing to persistent post-antibiotic GI dysfunction. Further, bloating and abdominal discomfort after antibiotic usage may occur via visceral hypersensitivity secondary to impaired gut barrier function, driven by depleted microbiome species and associated metabolites [7,8].

The mechanisms that shape recovery of the microbiome and gut barrier function after antibiotics are increasingly understood. Delayed or incomplete recovery of key microbial functions can perpetuate GI consequences [9,10]. Among the most critical functions lost during broad-spectrum antibiotic exposure is short-chain fatty acid biosynthesis, particularly of butyrate and acetate, which fuels epithelial repair processes and contributes to immune and barrier signaling within the colon [11,12,13]. In parallel, antibiotic-induced depletion of polyphenol-metabolizing microbes disrupts formation of downstream bioactive metabolites such as Urolithin A (UroA), which contributes to mucus layer maintenance and epithelial tight junction expression [14,15]. Delayed restoration of these metabolic pathways may therefore prolong epithelial vulnerability following antibiotic exposure.

Importantly, antibiotics also reshape competitive microbial networks in ways that may favor the expansion of pathobionts and the accumulation of host-toxic microbial metabolites during the recovery phase. Suppression of beneficial fermentative taxa can permit disproportionate regrowth of pro-inflammatory or barrier-disruptive organisms, while microbial metabolites such as *p*-cresol sulfate may rise during late recovery [16,17]. Overall, delayed recovery trajectories of beneficial microbiome community structure and function after antibiotics can lead to disrupted gut barrier integrity, and, in turn, acute and persistent GI symptoms.

Probiotics have emerged as promising interventions to mitigate microbiome perturbations and improve recovery from antibiotics [18,19,20]. Clinical evidence suggests that some specific strains may partially promote the recovery of commensal taxa and improve clinical outcomes [3,21,22,23,24]. For example, a recent meta-analysis suggests probiotic intervention after antibiotics may help preserve microbiome community structure [25]. However, results from probiotics trials are heterogenous given underlying biological differences in strains and formulation. Importantly, most prior probiotics trials have focused on clinical symptoms without elucidating the biological recovery of microbial function, metabolite output, and intestinal barrier physiology that mechanistically drive post-antibiotic GI outcomes [26,27]. Notably, a well-cited, mechanism-focused clinical trial by Suez and colleagues assessed 21 healthy adults treated with oral antibiotics (ciprofloxacin and metronidazole) for 7 days. The trial included eight participants who received a probiotics formulation and seven participants who received no intervention, both for 4 weeks following antibiotics. The authors reported that this formulation delayed the recovery of the baseline microbiome after antibiotics, noting however that the clinical efficacy of this specific formulation remains a subject of ongoing discussion [28,29].

Synbiotics, defined as “a mixture comprising live microorganisms and substrate(s) selectively utilized by host microorganisms that confers a health benefit on the host” [30], may have a greater physiological effect than probiotics alone. A meta-analysis of oral synbiotics demonstrates meaningful microbiome and microbiome metabolite modulation [31]. Additionally, recent preclinical data demonstrate that following antibiotics, probiotic microbes formulated with a polyphenol-based prebiotic accelerate recovery of beneficial bacteria such as butyrate- and UroA-producing microbes and increase both butyrate and acetate [32]. Subsequently, these synbiotic components were assessed in a randomized, placebo-controlled trial of healthy adults without antibiotic exposure and demonstrated mechanistic improvements, including an overall increase in key beneficial microbes, UroA production, butyrate production, and a correlation with a reduction in circulating CRP [33].

While Suez and colleagues [29] suggested potential biological risks, specifically delayed microbiome recovery when using probiotics after antibiotics, it remains unknown whether this synbiotic promotes restoration. This synbiotic was designed for functional recovery and demonstrated benefit in both preclinical models and a concurrent antibiotic-naïve population [33]. In parallel, we conducted a randomized, placebo-controlled clinical trial in healthy adults undergoing a standardized course of oral antibiotics (ciprofloxacin and metronidazole) to evaluate a 24-strain multi-species synbiotic with a polyphenol-based prebiotic using capsule-in-capsule delivery technology. These antibiotics were selected given their widespread use among clinicians, ability to induce a strong and consistent microbiome perturbation, and alignment with antibiotic regimens in previous probiotic mechanistic studies [29].

This study was designed as a proof-of-mechanism trial, an investigation intended to assess whether an intervention modulates key biological pathways and mechanistic endpoints. The aim of the clinical trial was to determine whether synbiotic administration during and after antibiotic exposure could accelerate restoration of microbiome diversity, recovery of beneficial metabolic outputs, suppression of detrimental microbial metabolites, and normalization of gut barrier integrity during the post-antibiotic recovery window.

## 2. Results

### 2.1. Participant Demographics

Table 1 shows participant demographics at baseline.

### 2.2. Microbiome Profile

**Figure 1:**
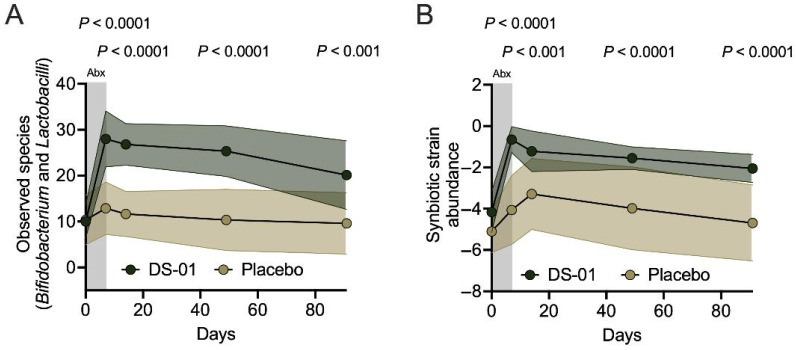
A multi-species synbiotic mediates fecal microbiome composition during and after antibiotics. Healthy adults underwent 7 days of oral broad-spectrum antibiotics (ciprofloxacin and metronidazole) and daily administration of a multi-species synbiotic or placebo: (**A**) Observed species over time between arms, measured by alpha-diversity of beneficial microbes (combined *Bifidobacterium* and *Lactobacillus* species), as measured through taxonomic profiling of stool metagenomes. (**B**) Synbiotic strain abundance, or the summed abundance of all detected synbiotic strains within the stool (total combined abundance of all synbiotic strain genomes), measured with the same method. Standard deviation of the arm was plotted with error polygons. Significance was measured between arms using linear mixed modeling.

Microbiome profiles were characterized using diversity and other microbiome-specific metrics including alpha-diversity of *Bifidobacterium* and *Lactobacillus* species, the abundance of synbiotic strains, and abundance and presence of native species. The alpha-diversity of *Bifidobacterium* and *Lactobacillus* species was significantly higher in the multi-species synbiotic arm immediately following cessation of antibiotics (Day 7, 2.1-fold-change, 117% difference, *p* < 0.0001) and during recovery from antibiotics (Day 14, 2.3-fold-change, 130% difference, *p* < 0.0001; Day 49, 2.4-fold-change, 144% difference, *p* < 0.0001; Day 91, 2.1-fold-change, 109% difference, *p* < 0.001) (Figure 1A). Additionally, the multi-species synbiotic arm had a significant increase in the abundance of synbiotic strains in the stool compared to placebo immediately following cessation of antibiotics (Day 7, 2587-fold-change, *p* < 0.0001) and during recovery from antibiotics (Day 14, 118-fold-change, *p* < 0.001; Day 49, 268-fold-change, *p* < 0.0001; Day 91, 435-fold-change, *p* < 0.0001) (Figure 1B). Notably, these high fold-changes reflect the low level of these strains in the placebo arm and the high precision of the detection method.

**Figure 2:**
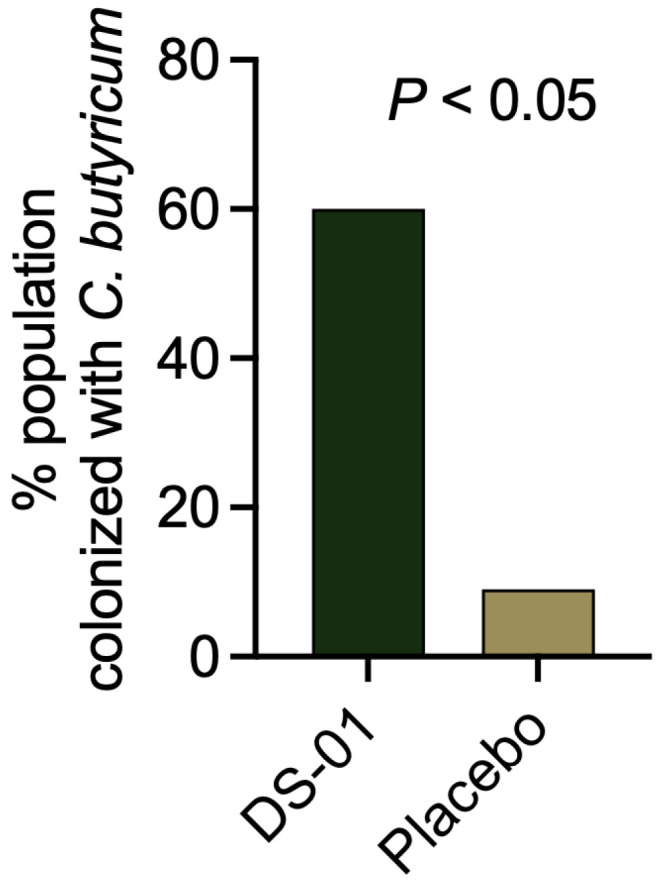
A multi-species synbiotic rapidly increases fecal *C. butyricum* colonization after antibiotics. Healthy adults underwent 7 days of oral broad-spectrum antibiotics (ciprofloxacin and metronidazole) and daily administration of a multi-species synbiotic or placebo. Presence or absence of *C. butyricum* colonization was assessed in the stool by enumerating presence of genomes at Day 14. Presence was detected in the sample if either (1) 1% of total k-mers and >2% of unique k-mers were present, or (2) >55% of total k-mers were present. Significance was measured between arms using Fisher’s exact test.

Next, we assessed the impact on native butyrate-producing bacteria in the stool. Specifically, the multi-species synbiotic significantly increased the abundance (23-fold-change, *p* < 0.05) and colonization (60% vs. 9%, *p* < 0.05) of the well-known native butyrate-producing microbe *Clostridium butyricum* compared to placebo (Figure 2). Interestingly, *C. butyricum* was not detectable in either study arm at baseline, suggesting supplementation with this multi-species synbiotic concurrent with antibiotics facilitated the colonization of this key butyrate-producing species. Further, the multi-species synbiotic rapidly increased other butyrate-producing microbes, including species from the genera *Roseburia* (8-fold-change, *p* < 0.05, Day 14) compared to placebo.

In addition, the multi-species synbiotic conferred a significant increase in the abundance of native butyrate-producing bacteria during long-term recovery from antibiotics, including a beneficial species from the genera *Clostridium_AA* (Day 49, 8-fold-change; Day 91, 6-fold-change, both *p* < 0.05). In the synbiotic arm, this represented a 7-fold increase in the abundance of a species from the genera *Clostridium_AA* after antibiotics (Day 0 to Day 91, *p* < 0.01).

**Figure 3:**
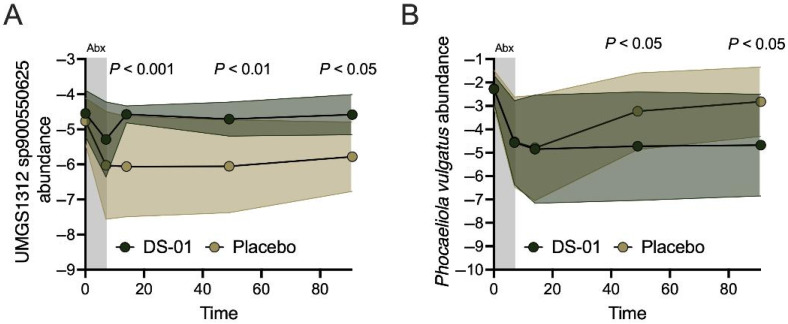
A multi-species synbiotic changes fecal abundance of key native microbes during recovery from antibiotics. Healthy adults underwent 7 days of oral broad-spectrum antibiotics (ciprofloxacin and metronidazole) and daily administration of a multi-species synbiotic or placebo. (**A**) Observed abundance of UMGS1312 sp900550625 or (**B**) *Phocaeicola vulgatus* during antibiotic treatment and recovery was enumerated. Standard deviation of the arm was plotted with error polygons. Significance was measured between arms using linear mixed modeling.

Importantly, native microbes were depleted during antibiotics (Figure 3A,B), including a novel beneficial microbe UMGS1312 sp900550625 from the *Oscillospiraceae* family. Recently, UMGS1312 sp900550625 was reported to be associated with protection against irritable bowel syndrome (IBS), sarcopenia, stress, and metabolic conditions (obesity, diabetes, obstructive sleep apnea, metabolic syndrome, dyslipidemia, hypertension, and metabolic dysfunction-associated liver disease) in a large cross-sectional study [34]. Importantly, the multi-species synbiotic significantly increased the recovery of the novel beneficial native species UMGS1312 sp900550625 after antibiotics compared to placebo, and the results persisted (Day 14, 31-fold-change, *p* < 0.001; Day 49, 22-fold-change, *p* < 0.01; Day 91, 16-fold-change, *p* < 0.05) (Figure 3A).

A native pathobiont, *Phocaeicola vulgatus* (formerly *Bacteroides vulgatus*), was decreased after antibiotics (Figure 3B). This potentially harmful species has been linked with decreased gut barrier function [35] and increased metabolic conditions (obesity, obstructive sleep apnea, diabetes, dyslipidemia, hypertension) in a large cross-sectional study [36]. The multi-species synbiotic significantly suppressed the recovery of *P. vulgatus* after antibiotics compared to placebo, and the result persisted (Day 49, −97% difference, *p* < 0.05; Day 91, −98% difference, *p* < 0.05) (Figure 3B).

### 2.3. Short-Chain Fatty Acids

SCFAs, including acetate and butyrate, are associated with decreased antibiotic-associated GI conditions [37]. The multi-species synbiotic facilitated the recovery of fecal acetate production during recovery from antibiotics, with a 62% restoration of acetate observed after antibiotic cessation through Day 91 (*p* < 0.01). In contrast, the placebo arm did not significantly affect fecal acetate production over the same period (*p* = NS).

Further, it has been documented in previous clinical trials that specific synbiotics are most effective in increasing fecal butyrate production in individuals with low baseline butyrate levels [33]. Thus, we evaluated the recovery of butyrate production after antibiotics in a population with low-baseline fecal butyrate. Similarly, we found that in this population the multi-species synbiotic promoted recovery of butyrate production, with a 119% increase after antibiotics through Day 91 (*p* < 0.05). In contrast, placebo did not affect butyrate production over the same period (*p* = NS).

### 2.4. Urolithin A

**Figure 4:**
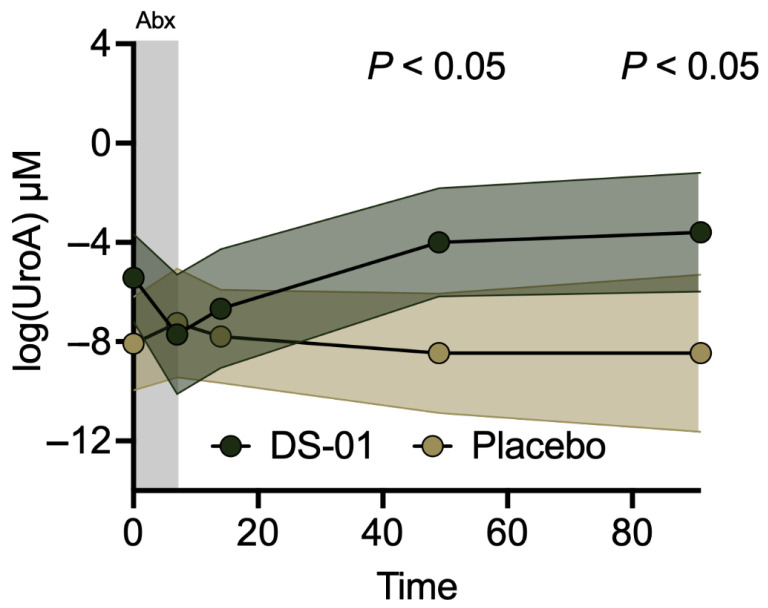
A multi-species synbiotic increases UroA production after antibiotics. Healthy adults underwent 7 days of oral broad-spectrum antibiotics (ciprofloxacin and metronidazole) and daily administration of a multi-species synbiotic or placebo. Urinary UroA (μM) was measured via mass spectrometry (LC-MS/MS) during and after antibiotics. Error polygons indicate mean and 95% CI. Significance was measured between arms using linear mixed-effects modeling.

UroA increases mucin production and expression of tight junction proteins to enhance gut barrier function. The multi-species synbiotic significantly increased urinary UroA production compared to placebo throughout the recovery period from antibiotics (Day 49, 87-fold-change, *p* < 0.01; Day 91, 131-fold-change, 13,008% difference, *p* < 0.05). These large percentage changes reflect the low baseline and post-antibiotic urinary UroA concentrations observed in the placebo arm. Absolute UroA concentrations across all timepoints remained in the low micromolar range, emphasizing that the fold-changes represent biologically meaningful recovery from near-undetectable levels rather than supraphysiological exposure. Mean urinary UroA concentrations in the synbiotic arm increased from 0.0044 µM (0.0007, 0.0258 µM) at baseline to 0.0277 µM (0.0025, 0.3037 µM) at Day 91, whereas concentrations in the placebo arm remained near or below the limit of detection throughout the study. Within the multi-species synbiotic arm, UroA production increased 6-fold over baseline by the end-of-study (Day 91, *p* < 0.02) (Figure 4). Additionally, the multi-species synbiotic arm had 80% UroA-producers (defined as the population with detectable urinary UroA) compared to 0% in the placebo arm at Day 91.

**Figure 5:**
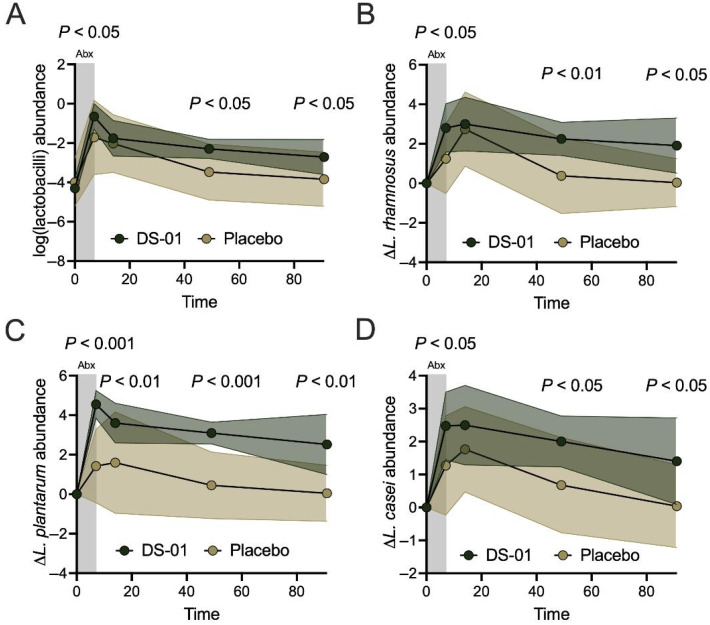
A multi-species synbiotic increases ellagic acid-producing *Lactobacillus* species abundance during and after antibiotics. Healthy adults underwent 7 days of oral broad-spectrum antibiotics (ciprofloxacin and metronidazole) and daily administration of a multi-species synbiotic or placebo. (**A**) The relative abundance of lactobacilli, defined here as the summed relative abundance of species within the genera *Lactobacillus*, *Ligilactobacillus*, *Lacticaseibacillus*, *Lactiplantibacillus*, and *Limosilactobacillus*, as measured through taxonomic profiling of stool metagenomes. The change in relative abundance of (**B**) *Lactobacillus rhamnosus*, (**C**) *Lactobacillus plantarum*, and (**D**) *Lacticaseibacillus casei* measured using the same method. For (**A**–**D**), standard deviation of the arm was plotted with error polygons and significance was measured between arms using linear mixed-effects modeling.

Mechanistically, some *Lactobacillus* species have been shown to metabolize ellagitannins into the UroA precursor ellagic acid. Microbiome analysis demonstrated the mean overall abundance of all detected lactobacilli was significantly higher in participants in the multi-species synbiotic arm compared to those in the placebo arm during antibiotics (Day 7, 11-fold-change, *p* < 0.05) and throughout recovery (Day 49, 15-fold-change, *p* < 0.05; Day 91, 13-fold-change, *p* < 0.05) (Figure 5A). There are three *Lactobacillus* species within the multi-species synbiotic that have been shown *in vitro* to produce ellagic acid from ellagitannins (*Lacticaseibacillus rhamnosus*, *Lactiplantibacillus plantarum*, and *Lacticaseibacillus casei*).

Overall, all three species showed an increase in the synbiotic arm. Specifically, *L. rhamnosus* was significantly increased at Day 7 (36-fold-change, *p* < 0.05), Day 49 (74-fold-change, *p* < 0.01), and Day 91 (75-fold-change, *p* < 0.05) compared to placebo. *L. plantarum* was significantly increased at Day 7 (1337-fold-change, *p* < 0.001), Day 14 (100-fold-change, *p* < 0.01), Day 49 (442-fold-change, *p* < 0.001), and Day 91 (298-fold-change, *p* < 0.01) compared to placebo. Lastly, *L. casei* was significantly increased at Day 7 (16-fold-change, *p* < 0.05), Day 49 (21-fold-change, *p* < 0.05), and Day 91 (23-fold-change, *p* < 0.05) compared to placebo (Figure 5B,C).

Notably, the abundance of UroA-producing *Ellagibacter* genera was significantly increased in participants in the synbiotic arm after antibiotics (Day 14, 20-fold-change, *p* < 0.01) compared to placebo. Furthermore, *Ellagibacter*, *Gordonibacter*, and *Enterocloster* abundance in the stool was positively correlated with UroA among multi-species synbiotic arm participants (*p* < 0.05 for all three genera).

### 2.5. Deleterious Microbiome-Derived Metabolites

**Figure 6:**
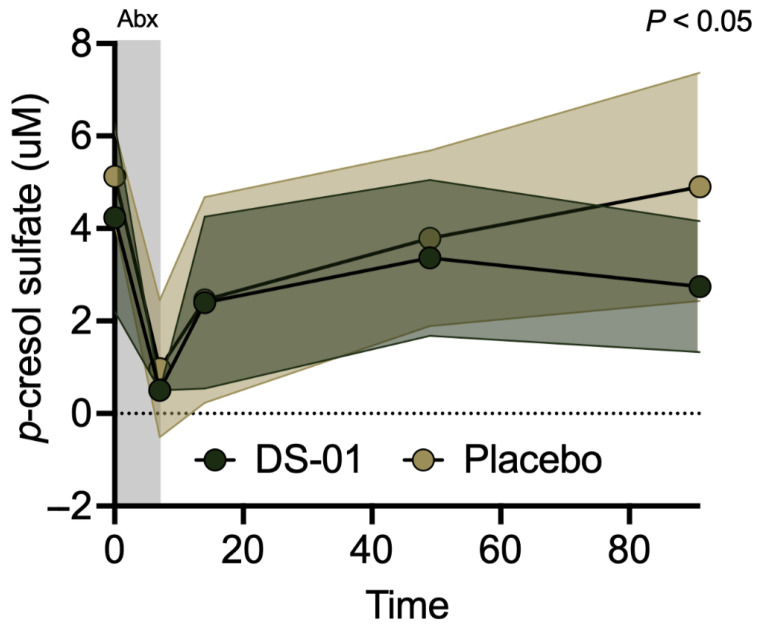
A multi-species synbiotic decreases serum *p*-cresol sulfate (pCS) after antibiotics. Healthy adults underwent 7 days of oral broad-spectrum antibiotics (ciprofloxacin and metronidazole) and daily administration of a multi-species synbiotic or placebo. pCS in the serum was measured via mass spectrometry (underivatized GC-MS) during and after antibiotics. Standard deviation of the arm was plotted with error polygons. Significance was measured between arms using linear mixed-effects modeling.

In both study arms, pCS in the serum nearly reached 0 uM after antibiotics (Day 7, *p* = NS, Figure 6). However, by Day 91, the multi-species synbiotic conferred a significant 68% decrease in serum pCS compared to placebo (*p* < 0.05, Figure 6). Mechanistically, synbiotic strain abundance was significantly correlated with diminished abundance of known *p*-cresol producer *Blautia hydrogenotrophica* (*p* < 0.01) in the multi-species synbiotic arm throughout recovery from antibiotics, and this relationship was not significant in the placebo arm. Further, the multi-species synbiotic transiently, but significantly, decreased the abundance of another cardiovascular-associated microbiome-derived uremic toxin, trimethylamine N-oxide (TMAO) at Day 49 (*p* < 0.05) compared to placebo.

### 2.6. Gut Barrier Integrity

**Figure 7:**
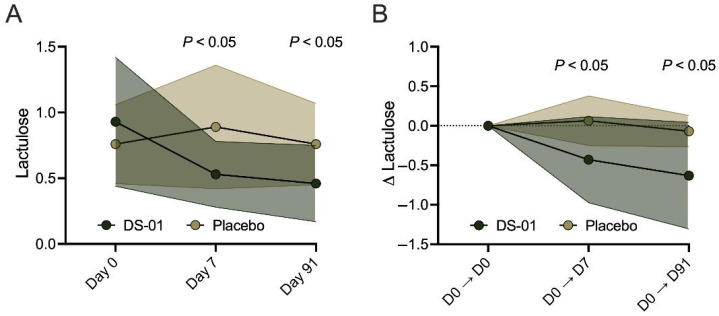
A multi-species synbiotic increases gut barrier integrity during and after antibiotics. Healthy adults underwent 7 days of oral broad-spectrum antibiotics (ciprofloxacin and metronidazole) and daily administration of a multi-species synbiotic or placebo. Gut barrier integrity was measured via mass spectrometry of the (**A**) urinary secretion of lactulose after the lactulose-based test and after antibiotics and synbiotic supplementation. (**B**) To calculate change from baseline, each timepoint has been derived from the equation Day X − Day 0. Standard deviation of the arm was plotted with error polygons. Significance was measured between arms using linear mixed modeling.

The impact of the multi-species synbiotic on the change in gut permeability during and after antibiotics was assessed using a lactulose-based test. Specifically, the percent recovery of urinary lactulose, a disaccharide that is non-metabolizable or absorbable by humans, was calculated after ingestion at baseline (Day 0) compared to study timepoints (Day 7 and Day 91). When measuring absolute lactulose recovery, the multi-species synbiotic increases gut barrier integrity by ~50% compared to placebo both during (Day 7, 51%, *p* < 0.05) and after (Day 91, 49%, *p* < 0.05) antibiotics (Figure 7A). Additionally, when measuring the change in lactulose from baseline, the multi-species synbiotic increases gut barrier integrity both during (Day 7, 305%, *p* < 0.05) and after (Day 91, 161%, *p* < 0.05) antibiotics compared to placebo (Figure 7B).

### 2.7. Safety

The multi-species synbiotic was well-tolerated, with no adverse events reported in either arm, including no serious adverse events. Clinical chemistry and hematology parameters showed no clinically relevant differences between study arms, and within-arm variations from baseline reflected normal physiological fluctuations. All values remained within reference ranges, consistent with a healthy study population (Supplementary Materials Table S1).

## 3. Discussion

This randomized, placebo-controlled clinical trial is the first to evaluate the administration of a 24-strain multi-species synbiotic during and after antibiotics, a formulation previously shown to promote recovery of microbiome and key microbiome metabolites after antibiotics *in vitro* [32]. The trial addressed critical gaps by evaluating the effects of a synbiotic on gut microbiome composition and function as well as gut barrier integrity during and after antibiotics. Importantly, these specific synbiotic results are in contrast to the results from a mechanism-focused probiotic trial that was similarly designed. Specifically, Suez and colleagues reported delayed microbiome recovery among healthy participants who received a probiotic formulation following the same antibiotic regimen [29]. These differences may reflect important distinctions in strain composition and delivery technology, underscoring the heterogeneity of probiotic and synbiotic interventions on outcomes. Major strengths of our study are the use of relatively broad-spectrum antibiotics, multiple sample collections across time, and ultra-deep sequencing with 100 M read depth that augments high-resolution metabolomic and biomarker profiling to elucidate the mechanisms driving the effects of a multi-species synbiotic.

Unlike our prior synbiotic investigation conducted in healthy adults without antibiotic exposure [33], the present trial specifically interrogates microbiome-driven recovery mechanisms following antibiotic-induced ecological disruption. The findings from the present study align with, yet distinctively expand upon, our previous clinical results in healthy adults who did not receive antibiotics [33]. While both trials demonstrated that the multi-species synbiotic significantly enriched *Bifidobacterium* and *Lactobacillus* species diversity, increased UroA production, and enhanced fecal butyrate, the ecological mechanisms driving these outcomes and the magnitude of changes appear context dependent. In the previous unperturbed population, the multi-species synbiotic exerted an *augmentative* effect, optimizing an already stable system and correlating with reduced systemic inflammation. In contrast, the present trial reveals a *restorative* mechanism. Here, the synbiotic actively rescued specific beneficial microbes and their functions following an acute ecological disturbance from antibiotics while suppressing recovery of potentially harmful microbes. This distinction is most evident in the colonization dynamics of *C. butyricum*. In the previously published non-antibiotic population, this strain did not meaningfully colonize, likely due to colonization resistance from a more stable native community. Conversely, antibiotics in the current trial likely reduced this colonization resistance, creating an open niche that allowed for significant colonization and persistence. Furthermore, while healthy individuals showed benefits in general inflammatory markers such as CRP, this antibiotic-perturbed population highlighted the capacity of synbiotic intervention to suppress specific dysbiosis-associated markers, including the pathobiont *P. vulgatus* and the metabolite pCS.

Overall, there are several important microbiome insights that support mechanistic and clinical implications. This multi-species synbiotic includes a *L. rhamnosus* strain that has been shown to reduce diarrhea by biofilm formation that mechanically protects gut barrier physiology [5]. Additionally, this strain induces the expression of ion channels and aquaporins (SLC26A3, NHE3, and AQP4) required for luminal water-electrolyte homeostasis in a mouse model of antibiotic treatment [38]. A depletion of this *L. rhamnosus* strain may impair gut barrier integrity and osmotic homeostasis, in turn leading to antibiotic-associated diarrhea. Accordingly, an increase in this strain may have clinical benefit.

Further, the impact of this multi-species synbiotic on native microbes uniquely promotes colonization and increased abundance of specific beneficial microbes while dampening pathobionts. The significant increase in the colonization and abundance of the butyrate-producing species *C. butyricum* is particularly relevant for post-antibiotic sequelae [23,39]. *C. butyricum* has been shown to increase gut barrier integrity via several mechanisms, including tight junction protein optimization [40,41,42], and reduce symptom severity in patients with diarrhea-predominant IBS, which may arise following infection or antibiotic exposure [43]. Additionally, the synbiotic promoted the recovery of the beneficial microbe UMGS1312 sp900550625 (*Oscillospiraceae*), a specific microbial taxa that has been associated with a decreased incidence of IBS [27]. Conversely, the synbiotic prevented the recovery of the native pathobiont *P. vulgatus*, which secretes barrier-disrupting proteases [35]. Collectively, these shifts suggest the synbiotic may support the underpinning biological recovery pathways associated with the mitigation of post-antibiotic GI dysfunction.

Broadly, the results of the clinical trial align with previous evidence supporting the optimization of gut barrier function by this multi-species synbiotic via increased beneficial microbially derived metabolites [32]. First, the multi-species synbiotic significantly increased SCFA-producing microbes as well as acetate and butyrate, key functional microbiome-derived metabolites that support gut barrier integrity through mechanisms such as tight junction regulation and epithelial cell turnover [11,12,13]. Importantly, the presence of SCFA-producing microbes allows for the fermentation of dietary carbohydrates, which otherwise can accumulate in the colon after antibiotics, resulting in increased water in the lumen of the colon and subsequent diarrhea [5,6]. Second, the multi-species synbiotic increased UroA production and ellagic acid-metabolizing *Lactobacillus* species, both important to enhance gut barrier integrity via increased mucin production and tight junction protein expression [14,15]. These results align with a previous clinical trial using this synbiotic in healthy adults without antibiotics [33]. Further, the multi-species synbiotic reduced systemic levels of pCS, a recognized microbiome-derived uremic toxin and surrogate marker for gut barrier dysfunction [44]. Given that pCS is implicated in cardiovascular and renal complications [45], its reduction may have implications beyond GI function via gut barrier optimization. While this proof-of-mechanism trial was not designed to define clinically meaningful thresholds, the observed reduction in pCS represents a favorable biological modulation. Overall, these results indicate that this multi-species synbiotic promotes recovery of key biological processes while modulating the reconstitution of potentially harmful taxa and metabolites.

Biologically, gut barrier function was evaluated using a gold-standard lactulose-based test. Overall, the multi-species synbiotic significantly enhanced gut barrier function both during (Day 7) and after (Day 91) antibiotics compared to placebo. This finding is notable given a meta-analysis of probiotics (517 participants across eight trials) reported other probiotics were unable to demonstrate an improvement in gut barrier function based on a lactulose-based test [46]. In contrast, participants in the present trial exhibited up to 305% improvement in intestinal permeability, an accepted functional surrogate for gut barrier function, compared to placebo. These data underscore the distinct effect of this synbiotic on reducing intestinal permeability, a key factor in GI symptoms.

As the focus of the trial was validating mechanistic insights, it was conducted in a healthy population with standardized antibiotics. Accordingly, this design choice leads to several limitations. This trial focused on mechanistic endpoints and did not include patient-reported GI symptom outcomes. While the observed improvements in microbiome composition, metabolite recovery, and gut barrier integrity provide strong biological plausibility for clinical benefit, future trials should incorporate validated symptom-based endpoints to directly link these mechanistic effects to clinical outcomes. In terms of generalizability, these results may not translate directly to individuals who receive a different antibiotic regimen, although these antibiotics induce robust dysbiosis. Similarly, participants in this study were healthy adults without baseline GI or underlying clinical conditions, so the findings may not be directly generalizable to populations at the highest risk for antibiotic-associated complications, including older adults, immunocompromised individuals, or patients with preexisting gut dysbiosis; consequently, future studies evaluating broader populations including clinically vulnerable participants may be warranted. Furthermore, the sample size was small with a higher attrition rate, which may limit generalizability. While this attrition may be due to the practical burden of the lactulose-based test, importantly, there was no difference in safety metrics between study arms. However, despite the modest study size, the sample is larger than that of a similar mechanism-focused trial [29] and the effect sizes for key biological endpoints were significant, demonstrating a robust biological signal. Additionally, variability in diet has the potential to influence the microbiome and its metabolites; however, this was not an inpatient study, and participants were instructed to strictly avoid fermented foods, probiotics, and other microbiome-modulating products to minimize variability. Lastly, further studies using a combination therapy of a synbiotic and complementary bioactive dietary compounds that further optimize SCFA production and gut barrier function (e.g., quercetin [47], spermidine [48,49], ginseng [50] and vitamin C [51]) may also be beneficial.

Despite the limitations, the data from this trial provide compelling evidence that a 24-strain synbiotic supports the preservation and recovery of microbiome community structure and key microbiome functions while promoting gut barrier function following antibiotics. These effects were evident during the acute antibiotic phase and persisted throughout the recovery period. In contrast to previous studies that question the utility and raised concerns about delays in microbiome recovery due to probiotic use, this trial suggests that this synbiotic may offer distinct advantages.

## 4. Materials and Methods

### 4.1. Clinical Trial Design

From an oversight perspective, Advarra IRB (Columbia, MD, USA) granted ethical clearance for this clinical trial and the procedures herein adhered to principles outlined in the Declaration of Helsinki. This study was executed at a single-site and conducted among healthy adult volunteers by an independent clinical research organization (KGK Science, London, ON, Canada) who conducted all clinical operations activities. The design was as a parallel, randomized, double-blind, placebo-controlled clinical trial. Written informed consent was obtained, and the participants were randomly assigned to receive either a multi-species synbiotic or a matching placebo for 91 consecutive days. Study products (identical in appearance to participants) were provided in capsule-in-capsule format. To induce a standardized dysbiosis challenge, all participants received broad-spectrum antibiotics during the first 7 days of the intervention period. The study endpoints included microbiome compositional dynamics assessed by whole-genome shotgun sequencing, fecal SCFAs, urinary UroA production, serum pCS and TMAO, gut barrier integrity assessed by a lactulose-based test, and safety parameters (vital signs, clinical chemistry, and hematology). Standard randomization, allocation concealment, and blinding procedures were maintained throughout the study. Data collection and laboratory analyses were performed by independent third-party laboratories.

**Figure 8:**
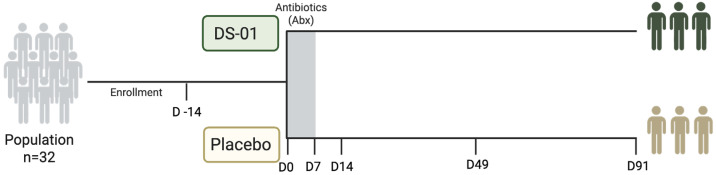
Overview of clinical trial design. Stool collection for metagenomics and metabolomics. Urine collection for UroA analysis on days 0, 7, 14, 49, and 91, and pregnancy test for female participants on days-14, 0, and 91. Twelve-hour fasted blood sample analysis on days 0, 7, 14, 49, and 91, and safety markers on days -14 and 91. Note: Study materials were dispensed on days 0, 7, 14, and 49; empty study material packaging was returned on days 7, 14, 49, and 91.

Figure 8 shows the clinical trial overview. Baseline 3-day food record, stool, urine, blood, and vital signs (resting HR, BP, weight) were collected. Participants repeated this procedure after 7, 14, 49 and 91 days.

### 4.2. Study Participants

Healthy males and females aged 18–55 years, with BMI 18.5–29.9 kg/m^2^, waist circumference <102 cm (men) or <88 cm (women), and normal medical evaluation were eligible. Participants had to agree to study restrictions, which included avoiding probiotics, fermented foods, NSAIDs, and certain drugs or supplements, while maintaining their usual level of physical activity. Key exclusions included recent antibiotic or probiotic use, chronic GI disease, autoimmune conditions, metabolic disease, pregnancy, and use of medications known to affect gut microbiota. Recruitment was conducted through local advertising and electronic postings. Eligible individuals completed baseline assessments before randomization.

**Figure 9:**
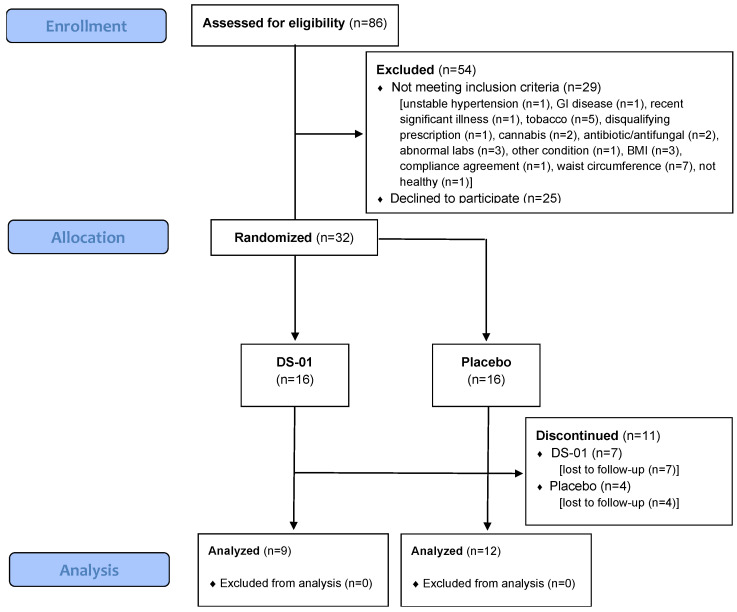
Consolidated Standards of Reporting Trials (CONSORT) flow chart for recruitment, allocation, and analysis of the study arms.

Figure 9 presents the Consolidated Standards of Reporting Trials (CONSORT) flow diagram. Of the 86 individuals who responded to recruitment and underwent eligibility screening, 32 met the inclusion criteria. These participants provided informed consent and were randomized in a 1:1 ratio via standard software into the two study groups. Eleven participants were lost to follow-up (Placebo: n = 4; DS-01: n = 7), leaving 21 participants who completed the trial and were included in the statistical analysis.

### 4.3. Screening

Participants who met preliminary eligibility attended an in-person session to receive the study details and provide written informed consent. Those who satisfied the inclusion criteria underwent a health assessment that included measurement of height, weight, resting heart rate (HR), blood pressure (BP), and fasting blood analysis. Before scheduled follow-up visits, participants completed repeated 4-day dietary records (3 weekdays and 1 weekend day) using an online application (Libero, v6.11, Nutritics, Dublin, Ireland). During the intervention period, participants adhered to a controlled exclusion diet including microbiome-mediating products as previously detailed [33] and described in the Supplementary Materials.

### 4.4. Antibiotic and Multi-Species Synbiotic Intervention

Participants were assigned to receive a daily regimen comprising an oral multi-species synbiotic or matching placebo (rice flour). The multi-species synbiotic was formulated with a nested capsule-in-capsule delivery technology (ViaCap). It delivered 53.6 billion AFU of a multi-species probiotic containing 24 different bacterial strains combined with 400 mg of prebiotic Indian pomegranate (*Punica granatum*; standardized to >40% polyphenols) (DS-01, Seed Health, Inc., Venice, CA, USA). The full strain composition is detailed in the Supplementary Materials and previously published [32]. Strain selection employed bioinformatic analysis to optimize the consortium for genomic diversity and functional redundancy. Formulation design emphasized post-antibiotic functional restoration by pairing fermentative taxa that provide metabolic scaffolding (lactate and acetate producers) with polyphenol substrates that support recovery of native UroA-converting species. Rather than relying on a single strain for metabolite synthesis, the consortium is engineered to drive ecological recovery via network interactions. Previous validation of strains suggested enhanced gut barrier integrity [52,53,54,55,56,57,58,59,60]. Preclinical validation in a dynamic gut model inoculated with human microbiota demonstrated recovery of SCFA output following antibiotic or ethanol perturbation, including significant enhancement of butyrate relative to controls [27].

All participants were also administered a 7-day oral antibiotic course consisting of ciprofloxacin (500 mg, twice daily) and metronidazole (500 mg, three times daily). This combination of antibiotics was selected to induce a standardized, clinically relevant dysbiosis comparable to other mechanistic clinical trials [29]. In addition, participants were instructed to consume 2 capsules (synbiotic or placebo) once per day, immediately prior to their morning meal. Dosing commenced at baseline (Day 0) and continued each day throughout the study for 91 days. Adherence was monitored by quantifying unused investigational product returned to the laboratory. In the event of a missed dose, participants were instructed to resume the standard supplementation (2 capsules) the next day. Ciprofloxacin and metronidazole were selected to induce a robust and reproducible disruption of both facultative and obligate anaerobic microbial communities, providing a standardized dysbiosis challenge for mechanistic evaluation. This regimen produces a consistent ecological perturbation and has been widely utilized in microbiome recovery studies to benchmark restorative interventions. While the magnitude of dysbiosis varies across antibiotic classes, the fundamental biological processes targeted in this study, specifically the depletion of beneficial fermentative taxa and the suppression of SCFA production, are common consequences of antibiotic exposure. Therefore, restorative mechanistic insights are expected to be relevant across diverse antibiotic applications.

### 4.5. Vitals Signs

Height and weight were recorded. Resting heart rate and blood pressure were measured in an upright, seated position following a five-minute rest period, with heart rate determined by palpation of the radial artery according to standard procedures.

### 4.6. Blood Sampling

Blood specimens were obtained via standard venipuncture by qualified study personnel. Samples were collected in appropriate vacutainer tubes. Quantification of serum pCS and TMAO was performed using gas chromatography–mass spectrometry (GC-MS C18/HILIC at Arome Science Inc., in Farmington, CT 06032, USA).

### 4.7. Stool Sample Collection and Fecal Metabolomic and Metagenomic Analysis

Fecal samples were self-collected using kits provided, frozen after collection, and transported via cold-chain procedures. Metagenomic sequencing, DNA extraction, library preparation, and sequencing was conducted at the University of Maryland Genomics Core (Rockville, MD, USA). SCFAs were quantified via a validated method (Arome Science Inc., Farmington, CT 06032, USA) [33]. Taxonomic profiling and alpha-diversity was assessed as previously described in a healthy non-antibiotic-treated population [33].

Synbiotic strain abundance was observed two ways: (1) mapping reads against clustered synbiotic genomes alone to examine sensitivity, and (2) together with >600 additional genomes from the genera comprising the synbiotic consortium to examine specificity. For approach (1), the 24 synbiotic strain genomes were clustered, yielding 19 representatives. Reads from the genomes marked present (≥30% unique and ≥40% total k-mers detected) were summed and then divided by total sample reads to calculate relative abundance. With this approach, synbiotic strain abundance in the synbiotic arm is 175-fold compared to the placebo arm at the end of the study.

For approach (2), reported in the results, synbiotic strain abundance was assessed alongside >600 additional genomes from genera included in the synbiotic consortium to evaluate specificity. A total of 7106 genomes meeting defined criteria (GTDB R220; RefSeq; CheckM2 completeness ≥ 90%, contamination ≤ 5%; genera *Bifidobacterium*, *Lacticaseibacillus*, *Lactiplantibacillus*, *Lactobacillus*, *Limosilactobacillus*, and *Ligilactobacillus*) were clustered with 24 synbiotic strain genomes at 99.2% ANI into 643 strain-level groups, each represented by one genome. Representatives included 18 independent synbiotic strain genomes, 384 *Lactobacillus* strains, and 241 *Bifidobacterium* strains. Synbiotic strain genomes were considered present if ≥15% unique and ≥25% total k-mers were detected or if >90% of total k-mers were found. Relative abundance was calculated by normalizing genome reads to total sample reads. Colonization herein is defined as detection of a bacterial species genome at a post-antibiotic timepoint, meeting predefined k-mer presence thresholds, that was not detected at baseline. While this approach identifies species that were not in the baseline populations but were gained during recovery following antibiotics, this method may be limited in definitively distinguishing stable colonization at the indicated timepoints without a post-supplement washout period.

### 4.8. Gut Permeability Test

Participants arrived at the clinic following a 12 h fast, during which only water was permitted from 12 to 8 h prior to the visit. Women who were menstruating were asked to reschedule their appointment. A lactulose-based test was conducted at the clinic where participants ingested a premeasured solution containing lactulose (250 mg/mL; Osmolax, Square Pharmaceutical Ltd., Dhaka, Bangladesh) and mannitol (50 mg/mL; Sigma, St. Louis, MO, USA) at a dose of 2 mL/kg body weight. Intestinal permeability was assessed based on the urinary recovery of the sugar over the subsequent 6 h. Although the solution was administered at the clinic, urine collection was completed at home. Participants were permitted to consume fructose-free foods and were instructed to drink water hourly, beginning two hours after ingesting the solution. The total volume of urine collected was recorded, and a sample was shipped the following morning via overnight express to Genova Diagnostics (Asheville, NC, USA) for analysis.

### 4.9. Urine Collection

The first void on the morning of the study visits was collected and changes in UroA were assessed via mass spectrometry (LC-MS/MS) (Arome Science Inc., Farmington, CT, USA) [34].

### 4.10. Safety Evaluation

Safety outcomes were evaluated by continuous monitoring of vital signs and systematic adverse event surveillance throughout the intervention. Comprehensive clinical chemistry and hematology panels were obtained at screening and study completion to assess hepatic, renal, metabolic, and hematologic status. Safety endpoints were analyzed descriptively, and between-group comparisons were performed where appropriate.

### 4.11. Statistical Analysis

Statistical analyses were performed using R (v4.4.3, [61]). Linear mixed-effects models were run using the packages ‘lme4’ or ‘nlme’. Longitudinal changes in microbiome, metabolite, and gut permeability endpoints were analyzed using mixed modeling frameworks incorporating fixed effects for treatment group, time, and their interaction, with random intercepts for participants to account for repeated measures. *C. butyricum* colonization was determined using Fisher’s exact test. Distributional assumptions were examined graphically and analytically. Where appropriate, outcome variables were transformed or variance structures modeled to maintain statistical validity. Categorical microbiome features were evaluated using exact tests. Significance was defined a priori at *p* < 0.05.

## 5. Conclusions

In this randomized controlled trial, administration of a multi-species synbiotic during and following broad-spectrum antibiotic exposure accelerated restoration of microbiome diversity, promoted recovery of key microbial metabolic outputs, and improved indices of gut barrier function, outcomes mechanistically linked to post-antibiotic recovery.

## Figures and Tables

**Table 1:** Participant characteristics.

	All (n = 32)		Synbiotic (n = 16)		Placebo (n = 16)	
	Mean	SD	Mean	SD	Mean	SD
Age (years)	31.2	7.9	33.3	9.0	29.1	6.1
Sex (% female)	62.5	NA	75	NA	50	NA
Height (cm)	168.1	11.4	165.4	11.2	170.8	11.3
Body Weight (kg)	68.7	12.0	66.6	12.6	70.8	11.3
BMI (kg/m^2^)	24.2	2.8	24.2	2.9	24.2	2.8

## Data Availability

Data and statistical analyses are available for non-commercial scientific inquiry and/or educational if requested and their use does not violate IRB restrictions and/or research agreement terms.

## Supplementary Materials

The following supporting information can be downloaded at: https://www.mdpi.com/article/10.3390/antibiotics15020138/s1, Table S1: Changes in safety markers from screening to end of study.

## Abbreviations

The following abbreviations are used in this manuscript:

AFU	Active Fluorescent Units
pCS	*p*-cresol sulfate
UroA	Urolithin A
