# Supplementary material for: Multi-Species Synbiotic Supplementation After Antibiotics Promotes Recovery of Microbial Diversity and Function, and Increases Gut Barrier Integrity: A Randomized, Placebo-Controlled Trial
Source: Antibiotics (Basel). 2026 Jan 30;15(2):138. doi: 10.3390/antibiotics15020138 (PMC12937403; doi:10.3390/antibiotics15020138)
Supplement: Supplementary file 1 [file antibiotics-15-00138-s001.zip › antibiotics-4074305-supplementary.pdf]

Supplement

Table S1. Changes in safety markers from screening to end of study. Hba1c: Hemoglobin A1c; eGFR: estimated glomerular filtration rate; AST: Aspartate Aminotransferase; ALT: Alanine Aminotransferase; RBC: Red Blood Cell count; MCV: Mean Corpuscular Volume; MCH: Mean Corpuscular Hemoglobin; MCHC: Mean Corpuscular Hemoglobin Concentration; RDW: Red Cell Distribution Width; WBC: White Blood Cell; MPV: Mean Platelet Volume.

**Variable**	**Study Arm**	**Day 0**	**Day 91**	***p*-value**	***p*-value**
		Mean ± SD	Mean ± SD	Within Arms	Between Arms
Bilirubin (log(μmol/L))	Placebo	2.2 ± 0.3	2.3 ± 0.2	0.91	0.75 (Day 0)
	Synbiotic	2.3 ± 0.5	2.1 ± 0.5	0.16	0.37 (Day 91)
Creatinine (μmol/L)	Placebo	78.6 ± 11.8	79.1 ± 9.7	0.17	0.07 (Day 0)
	Synbiotic	68.0 ± 18.8	67.8 ± 22.7	0.69	0.04 (Day 91)
Glucose (mmol/L)	Placebo	4.9 ± 0.3	4.9 ± 0.3	0.54	0.16 (Day 0)
	Synbiotic	5.0 ± 0.5	5.0 ± 0.3	0.87	0.53 (Day 91)
HbA1c (%)	Placebo	5.2 ± 0.2	5.3 ± 0.2	0.65	0.02 (Day 0)
	Synbiotic	5.4 ± 0.3	5.3 ± 0.2	0.82	0.04 (Day 91)
eGFR (mL/min/1.73m^2)	Placebo	102.7 ± 15.4	99.6 ± 12.3	0.07	0.63 (Day 0)
	Synbiotic	105.6 ± 19.6	105.3 ± 18.0	0.37	0.40 (Day 91)
Sodium (mmol/L)	Placebo	139.8 ± 2.3	140.8 ± 1.5	0.29	0.87 (Day 0)
	Synbiotic	139.6 ± 1.7	138.7 ± 3.2	0.22	0.11 (Day 91)
Potassium (mmol/L)	Placebo	4.6 ± 0.4	4.3 ± 0.3	0.07	0.52 (Day 0)
	Synbiotic	4.7 ± 0.4	4.4 ± 0.3	0.07	0.65 (Day 91)
Chloride (mmol/L)	Placebo	102.9 ± 1.8	103.2 ± 2.1	0.93	0.37 (Day 0)
	Synbiotic	103.7 ± 2.1	102.8 ± 3.8	0.21	0.93 (Day 91)
AST (U/L)	Placebo	20.9 ± 6.2	17.7 ± 2.4	0.03	0.12 (Day 0)
	Synbiotic	18.2 ± 4.1	17.4 ± 4.4	0.94	0.84 (Day 91)
ALT (U/L)	Placebo	18.7 ± 8.6	16.5 ± 5.8	0.43	0.23 (Day 0)
	Synbiotic	15.4 ± 6.8	14.9 ± 7.4	0.78	0.41 (Day 91)
Hemoglobin (g/L)	Placebo	139.8 ± 11.7	137.7 ± 8.8	0.69	0.22 (Day 0)
	Synbiotic	134.4 ± 13.1	134.3 ± 14.1	0.87	0.22 (Day 91)
Hematocrit (L/L)	Placebo	0.4 ± 0.0	0.4 ± 0.0	0.64	0.20 (Day 0)

	Synbiotic	0.4 ± 0.0	0.4 ± 0.0	0.64	0.26 (Day 91)
RBC (x1021/L)	Placebo	4.8 ± 0.7	4.8 ± 0.8	0.34	0.16 (Day 0)
	Synbiotic	4.5 ± 0.5	4.5 ± 0.5	0.97	0.10 (Day 91)
MCV (ft)	Placebo	88.1 ± 7.9	87.2 ± 9.1	0.95	0.43 (Day 0)
	Synbiotic	89.8 ± 3.2	88.4 ± 2.9	0.93	0.45 (Day 91)
MCH (pg)	Placebo	29.6 ± 3.1	29.0 ± 3.2	0.37	0.58 (Day 0)
	Synbiotic	30.1 ± 1.2	29.8 ± 1.0	0.64	0.51 (Day 91)
MCHC (g/L)	Placebo	335.2 ± 8.8	331.7 ± 8.6	0.28	0.80 (Day 0)
	Synbiotic	334.5 ± 6.8	335.3 ± 4.0	0.80	0.70 (Day 91)
RDW (%)	Placebo	13.2 ± 0.8	13.3 ± 1.1	0.44	0.35 (Day 0)
	Synbiotic	13.5 ± 0.9	13.7 ± 0.6	0.32	0.33 (Day 91)
WBC (log(x10^9/L))	Placebo	1.6 ± 0.2	1.6 ± 0.2	0.67	0.28 (Day 0)
	Synbiotic	1.7 ± 0.2	1.8 ± 0.3	0.29	0.18 (Day 91)
Platelets (x10^9/L)	Placebo	242.7 ± 51.0	246.0 ± 69.5	0.33	0.37 (Day 0)
	Synbiotic	261.7 ± 66.6	250.8 ± 56.9	0.16	0.65 (Day 91)
MPV (fl)	Placebo	8.9 ± 0.9	8.9 ± 1.3	0.35	0.43 (Day 0)
	Synbiotic	9.2 ± 0.9	9.2 ± 0.9	0.03	0.24 (Day 91)
Absolute Neutrophils (log(x10^9/L))	Placebo	1.0 ± 0.4	0.9 ± 0.4	0.84	0.19 (Day 0)
	Synbiotic	1.2 ± 0.3	1.2 ± 0.3	0.49	0.16 (Day 91)
Absolute Lymphocytes (x10^9/L)	Placebo	1.6 ± 0.3	1.6 ± 0.2	0.70	0.58 (Day 0)
	Synbiotic	1.6 ± 0.3	1.8 ± 0.5	0.23	0.29 (Day 91)
Absolute Monocytes (x10^9/L)	Placebo	0.4 ± 0.2	0.4 ± 0.0	0.70	0.52 (Day 0)
	Synbiotic	0.4 ± 0.1	0.4 ± 0.2	0.17	0.94(Day 91)
Absolute Eosinophils (x10^9/L)	Placebo	0.1 ± 0.1	0.1 ± 0.1	0.82	0.59 (Day 0)
	Synbiotic	0.1 ± 0.1	0.1 ± 0.1	0.18	0.70 (Day 91)
Absolute Basophils (x10^9/L)	Placebo	0.0 ± 0.0	0.0 ± 0.0	1.00	0.23 (Day 0)
	Synbiotic	0.0 ± 0.0	0.0 ± 0.0	0.36	1.00 (Day 91)

Controlled exclusion diet

Throughout the trial, participants were required to avoid foods and beverages containing live bacteria or yeast, including probiotic supplements, yogurt, fermented vegetables (e.g., sauerkraut, kimchi, pickles), fermented soy products (e.g., tempeh, miso, natto), uncooked buttermilk, soft or live-culture cheeses, raw sausages, and any

products labeled “Probiotic” or “Live Culture.” Restricted beverages included kefir, kombucha, apple cider vinegar, lambic beers, sour ales, wheat beers, and other probiotic-labeled drinks. Dietary records were reviewed by trained staff at each visit, and counseling was provided as necessary. To ensure consistency, participants were asked to replicate the exclusionary diet before and after each testing session.

Strain composition

Bifidobacterium longum SD-BB536-JP, *Bifidobacterium breve* SD-BR3-IT, *Lactiplantibacillus plantarum* SD-LP1-IT, *Lacticaseibacillus rhamnosus* SD-LR6-IT, *Lacticaseibacillus rhamnosus* HRVD113-US, *Bifidobacterium infantis* SD-M63-JP, *Bifidobacterium lactis* SD-BS5-IT, *Bifidobacterium lactis* HRVD524-US, *Lactobacillus crispatus* SD-LCR01-IT, *Lacticaseibacillus casei* HRVD300-US, *Bifidobacterium breve* HRVD521-US, *Bifidobacterium longum* HRVD90b-US, *Bifidobacterium lactis* SD150-BE, *Limosilactobacillus fermentum* SD-LF8-IT, *Lacticaseibacillus rhamnosus* SD-GG-BE, *Limosilactobacillus reuteri* RD830-FR, *Ligilactobacillus salivarius* SD-LS1-IT, *Bifidobacterium lactis* SD-CECT8145-SP, *Bifidobacterium longum* SD-CECT7347-SP, *Lacticaseibacillus casei* SD-CECT9104-SP, *Lactiplantibacillus plantarum* SD-LPLDL-UK, *Bifidobacterium lactis* SD-MB2409-IT, *Bifidobacterium adolescentis* SD-BA5-IT, and *Limosilactobacillus reuteri* SD-LRE2-IT (DS-01, Seed Health, Inc., Venice, CA, USA)
